# Evidence on the effectiveness of policies promoting price transparency - A systematic review

**DOI:** 10.1016/j.healthpol.2022.11.002

**Published:** 2023-08

**Authors:** Iris R. Joosse, David Tordrup, Julie Glanville, Eleanor Kotas, Aukje K. Mantel-Teeuwisse, Hendrika A. van den Ham

**Affiliations:** a Utrecht Centre for Pharmaceutical Policy and Regulation, Utrecht Institute for Pharmaceutical Sciences (UIPS), Utrecht University, Universiteitsweg 99, 3584 CG, Utrecht, the Netherlands; b York Health Economics Consortium (YHEC), York, YO10 5NQ, United Kingdom

**Keywords:** Price transparency, Systematic review, Pricing policy, Medicine prices

## Abstract

Policies promoting price transparency may be an important approach to control medicine prices and achieve better access to medicines. As part of a wider review, we aimed to systematically determine whether policies promoting price transparency are effective in managing the prices of pharmaceutical products. We searched for studies published between January 1, 2004 and October 10, 2019, comparing policies promoting price transparency against other interventions or a counterfactual. Eligible study designs included randomized trials, and non-randomized or quasi-experimental studies such as interrupted time-series (ITS), repeated measures (RM), and controlled before-after studies. Studies were eligible if they included at least one of the following outcomes: price (or expenditure as a proxy for price and volume), volume, availability or affordability of pharmaceutical products. The quality of the evidence was assessed using the GRADE methodology. A total of 32011 records were retrieved, two of which were eligible for inclusion. Although based on evidence from a single study, public disclosure of medicine prices may be effective in reducing prices of medicines short-term, with benefits possibly sustained long-term. Evidence on the impact of a cost-feedback approach to prescribers was inconclusive. No evidence was found for impact on the outcomes volume, availability or affordability. The overall lack of evidence on policies promoting price transparency is a clear call for further research.

## Highlights

• Research on policies promoting price transparency is scarce and (partially) inconclusive.
• Public disclosure of medicine prices could be effective in reducing prices.
• More robust evidence is needed to confirm the effects of these policies.
• Future research should also focus on unintended effects of these policies.

## 1 Introduction

In recent years, improved price transparency of pharmaceuticals has emerged as an important yet highly debated approach to manage medicine prices. This approach is believed to contribute to expanded access to medicines through the reduction of medicine prices [1]. In both the 2019 Fair Pricing Forum and the 72^nd^ World Health Assembly (WHA) the need for reliable information on medicine prices was emphasized, leading to the WHA's adoption of a resolution on advancing the transparency of markets for pharmaceuticals (WHA 72.8) [2,3].

The importance of promoting price transparency has also been reflected in various initiatives and regulations aiming to enhance transparent pricing. One such example is the Medicines Transparency Alliance (MeTA) initiative by the World Health Organization (WHO), which sought to develop national-level multistakeholder platforms to share data on the selection, procurement, quality, availability, pricing, promotion and use of medicines [4,5,6]. Another example is the European Union (EU) Transparency directive which requires the publication of the list prices of all reimbursable medicines in Europe [7].

The underlying rationale for promoting price transparency is that it may improve economic efficiency, as conventional economic theory indicates; assist policymakers and researchers through reliable price information; empower buyers to negotiate more strategically; increase accountability of manufacturers and governments for prices; and promote cost-effective decision-making by prescribers and patients [8,9]. Conversely, a lack of price transparency may give rise to corruption as confidential agreements may compromise accountability, especially in healthcare systems with weak governance [8,10]. These theories cut across four levels where transparency may occur: 1) the reporting of R&D and production costs, 2) the disclosure of net transaction prices to stakeholders as an input to price benchmarking, 3) the disclosure and control of prices along the supply chain, and 4) the communication of prices to prescribers or patients [8].

At the same time, there are concerns that improving transparency may lead to an increase in prices for lower-income countries, as manufacturers might abandon differential pricing schemes and apply uniform pricing for all countries to refrain from the appearance of unfair pricing [11]. Other harmful effects suggested are discouraged entry in poorer markets, reduced competition and lessened incentives for investments [11,12]. Despite the different claims that have been made, the impact of transparency measures on medicine accessibility remains largely theoretical thus far. It is, however, essential that governments and policy-makers implement measures that have proven to be effective. The 2015 WHO Guideline on Country Pharmaceutical Pricing Policies, which aimed to assist countries in evidence-based policy-making, did not include guidance on policies promoting price transparency [13]. The update of the 2020 Guideline therefore called for identification and assessment of the available evidence on price transparency measures, among nine other pricing policies [14]. Hence, the purpose of this systematic review is to determine whether policies promoting price transparency are effective in managing the prices of pharmaceutical products, with consideration to their impact on the volume, availability and affordability of these products. This review also aims to elucidate what contextual factors and implementation strategies may influence the effects of such policies.

## 2 Methods

This systematic review was undertaken according to the principles of systematic reviewing embodied in the Cochrane Handbook and guidance document published by the Centre for Reviews and Dissemination (CRD) [15,16]. The methodology and detailed search strategies have been described in detail previously [17,18].

As part of a wider review on ten pharmaceutical pricing policies, this paper only addresses policies promoting price transparency as a pricing approach.

### 2.1 Search strategy and selection criteria

An extensive literature search was performed between September 5 and October 10, 2019, for relevant articles published from 2004 to the search date in a large number of databases including but not limited to Ovid MEDLINE (Ovid), Embase (Ovid), Social Science Citation Index, EconLit, and the NHS Economic Evaluations Database (NHS EED). A variety of grey literature sources were also searched. The main structure of the search strategy comprised concepts pertaining to 1) non-specific pharmaceutical pricing policies (e.g. terminology related to pricing/prices combined with terms for medicines) or to 2) pharmaceuticals and one of ten specific pricing policies, among which were policies promoting price transparency (e.g. terminology related to pricing/prices combined with terms for transparency, including related terms such as disclosure, rebates, sharing, and accountability). Supplementary search approaches included reference-list checking and contacting experts.

### 2.2 Selection criteria

**Fig. 1:**
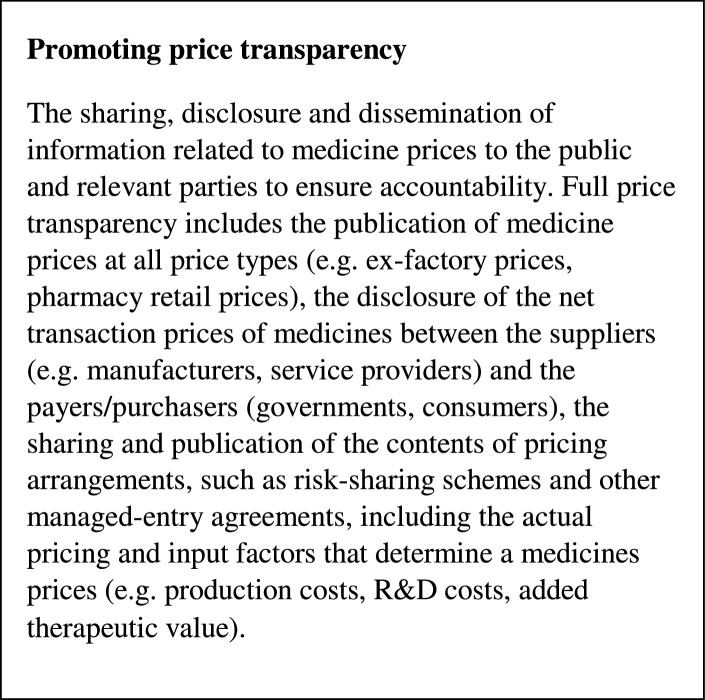
Definition of the policy intervention.

This systematic review only included studies that used robust experimental or observational study designs comparing policies promoting price transparency (see Fig. 1) to at least one comparator or counterfactual [15]. Study designs including randomized trials and non-randomized or quasi-experimental studies (including interrupted time-series (ITS), repeated measures (RM), panel data analyses, and controlled before-after studies) were considered strong designs. Single policies, or combinations of policies, were considered eligible. Studies reporting at least one of the primary outcomes of interest, i.e. price (or expenditure as a proxy), volume, availability or affordability, were eligible for inclusion. Medicine prices reported at all levels of the supply chain (e.g. ex-factory price, wholesale price, retail price, or patient price) were considered eligible. Outcomes in both public, private and mixed public-private settings were of interest.

### 2.3 Study selection

A single researcher assessed all titles and abstracts identified from the database searches and removed the obviously irrelevant records based on titles and abstracts. Two reviewers independently screened the titles and abstracts of potentially eligible records, with disagreements adjudicated by a third reviewer. The full texts of studies identified as potentially relevant were then subject to an eligibility check by two members of the review team independently (TB and CL or IRJ and HAvdH) before data extraction. Disagreements about study selection were resolved by discussion, and if consensus could not be reached, a third reviewer (DT or AKM-T) was consulted.

### 2.4 Data extraction and quality assessment

Data from included studies was extracted by one member of the review team (IRJ or LT) using a standardized data extraction form, including information on study design, setting and subjects, interventions including implementation strategies, outcomes, and results including contextual factors. Extracted data was verified by a second reviewer (HAvdH or DT) for accuracy.

The risk of bias in each included study was assessed by the extracting reviewer and checked by a second reviewer. Any disagreements were resolved by discussion until a consensus was reached. The assessment was done according to the EPOC guidelines, in which bias assessment criteria were adapted to study design [19]. Randomized and non-randomized trials and controlled before-after studies were assessed on nine criteria; ITS and RM studies were assessed on 8 criteria; and a set of four assessment criteria applied to all other study types. An explanation of the bias criteria is presented in Appendix 1.

The quality of the evidence was assessed by use of the GRADE methodology [20]. GRADE evidence levels were determined by considering the body of evidence available for each (sub-)intervention. Domains of scoring were the risk of bias, inconsistency of results, indirectness of evidence, imprecision of results, and ‘other’. Studies were upgraded in the ‘other’ domain if strong observational study designs were used (ITS, RM, panel data/regression analysis), according to precedent in literature [21]. The resultant certainty of the evidence was expressed as high, moderate, low or very low.

### 2.5 Data analysis

Substantial expected differences in the characteristics and contexts of included studies meant we did not aim to undertake a meta-analysis. Instead, we provided a narrative summary describing the quality of the studies, the relationship between interventions and patterns discerned in the data.

## 3 Results

**Fig. 2:**
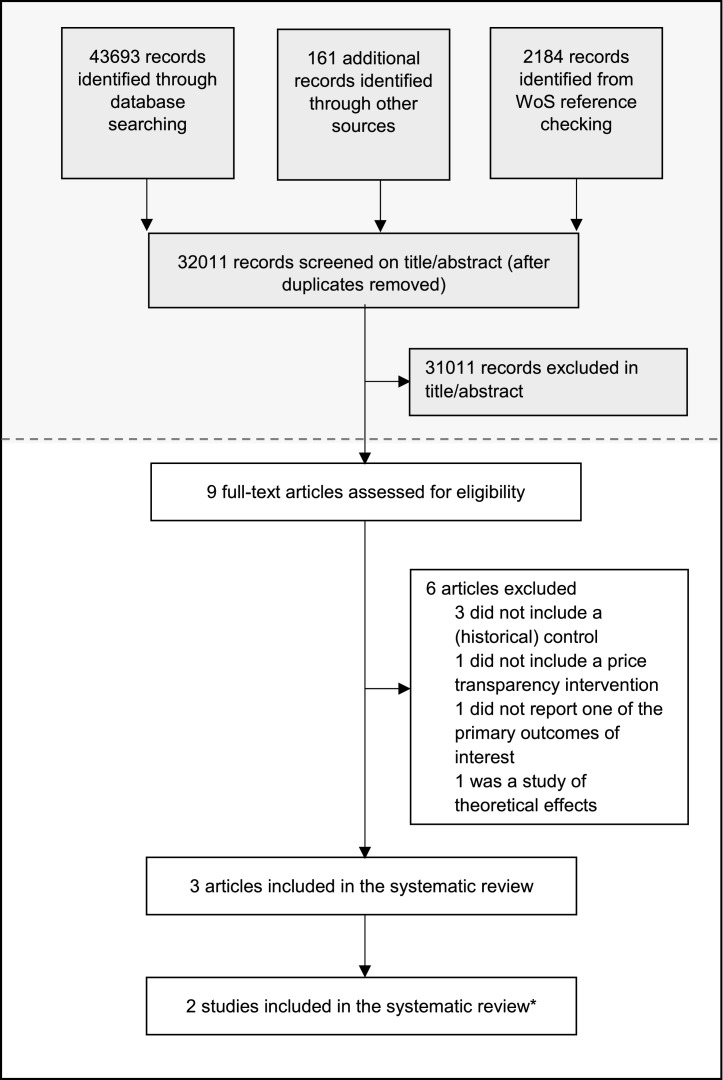
Flow chart of study selection. The number of articles identified through database searching and screening by title and abstract shown in grey apply to the overall search; as per protocol the database search included search terms for all ten specific pricing policies among which policies promoting price transparency was one. The lower part of the flow chart shown in white is specific to the selection of studies on policies promoting price transparency. WoS=Web of Science. *Two articles are part of the same study, but were published separately. These references are considered one study.

Electronic database and grey literature searches identified 43,693 records for all ten pricing policies combined. The review of relevant reference lists and other sources yielded a further 2,345 records. After removal of duplicates, 32,011 articles were screened by title and abstract, resulting in 1,000 potential articles to be included in the wider review. Nine of these articles were specific to policies promoting price transparency at first sight. After full-text screening, three scientific articles covering two policy measures were included in this section of the systematic review (Fig. 2). Specifically, two articles (Moodley 2019a, Moodley 2019b) are part of the same study, one addressing originator pharmaceuticals while the other addresses both originator and generic pharmaceuticals [22,23]. These references are considered to be one study in this review, according to Cochrane guidelines. Six studies were excluded, because of a lack of a historical control [24], [25], [26], primary outcomes of interest were not reported [27], theoretical effects were studied [28] and one study was considered off-topic after reading the full text [29].

Both studies identified had an interrupted time series design, one examining data from the United Kingdom [30] and one being set in the private sector in South Africa [22,23] (Table 1). Langley et al. included antibiotics and inhaled corticosteroids and examined the effects of a cost-feedback approach to prescribing physicians on drug expenditure. Moodley et al. considered the top 50 medicines in the private sector and examined the effects of mandatory public disclosure of medicine prices along the supply chain. The results were categorized according to their level of intervention.

**Table 1:** Summary of included studies.

	**Study type**	**Setting**	**Subjects of study**	**Intervention**	**Outcomes**
Langley 2018 et al. [30]	ITS	United Kingdom	Antibiotics and inhaled corticosteroids	A cost-feedback approach to prescribers: the provision of information on the cost of drugs in electronic prescribing to clinicians in a hospital setting	Price outcomes (weekly expenditure; weekly cost per patient)
Moodley 2019 et al. [22,23]	ITS	South Africa	Top-50 originator medicines dispensed in private sector by volume and corresponding generics	The Single-Exit-Price (SEP): mandatory disclosure of fixed medicine prices, that are composed of the weighted average of the sales price, the logistics fee and value-added taxes. All discounts and off-invoice rebates are removed. Applies to the private sector. The disclosed prices are made publicly available on the South African Medicine Price Registry website	Price outcome (relative change in medicine price)

ITS=Interrupted time series.

The results of the risk of bias assessment are presented in Table 2. The study by Langley et al. was associated with a low risk of bias across all domains, and overall certainty of evidence was assessed to be moderate. The study by Moodley et al. was associated with an unclear risk of bias across three of eight domains. None of these potential biases were considered to be of major influence on the results, and the overall risk of bias was thus considered to be low in this study. The certainty of evidence was assessed to be low for measures promoting public price disclosure due to serious indirectness. Detailed results of the overall quality assessment (GRADE) are provided in Appendix 3.

**Table 2:** Risk of bias assessment of included studies.

Bias type		Langley 2018	Moodley 2019†
Intervention independent	**ITS and RM studies**⁎		
Appropriate analysis			
Pre-specified shape of effect			
Intervention to affect data collection			
Incomplete outcome data	**All study types**		
Knowledge of allocated intervention			
Selective outcome reporting			
Other bias			

⁎ Bias domains only applicable to ITS and RM studies. ^†^Bias domains applicable to all study types. ITS=interrupted time series, RM=repeated measures.

† Moodley et al. [22] was assessed to have an unclear risk of bias across three domains due to the source of data not being described in detail (*intervention to affect data collection*), possible bias due to missing data (*incomplete outcome data*) and the outcome measure not being described in detail (*other bias*). The second reference [23] was similar to the first, except that the analysis method was not reported. However the two references are by the same authors, using the same dataset and methodology. As the analysis is appropriately reported in one of the studies (low risk of bias) but with less detail in the other (unclear risk of bias), it is reasonable to assume both studies are of equal quality. Overall the risk of bias is considered to be low for the two publications collectively.

### 3.1 Communicating prices to prescribers or patients

Langley et al. examined the impact of cost-feedback to prescribers in a hospital setting [30]. Clinicians were provided with extra information on the costs of drugs during prescribing, with the simple aim of informing them of the costs of their decision without intending to direct their prescription behavior. The intervention was implemented in November 2014 in the hospital's electronic prescribing system, which permitted the costs of the medicine of choice to be added to the display that the prescribing clinician sees immediately prior to selecting the drug.

The study reported expenditure outcomes for antibiotics and inhaled corticosteroids (Table 3). For antibiotics, a decrease of GBP -3.75 (p=0008) in weekly costs paid by the patient was observed immediately after implementation of the intervention, whereas the trend slightly increased with GBP 0.10 (p=0.015) over a twelve-month period. For inhaled corticosteroids, a small change in trend was seen in weekly costs per patient of GBP -0.03 (p=0.11), but no other changes were observed. The authors were not able to explain the contradictory results in both drug classes.

**Table 3:** Summary of findings of cost-feedback approaches to prescribers.

**Cost-feedback to prescribers compared to no cost-feedback approach**				
**Medicines:** Antibiotics and inhaled corticosteroids				
**Settings:** United Kingdom				
**Intervention:** Cost-feedback to prescribers				
**Comparison:** No policy				
**Outcomes**	**Impacts**	**No. of studies**	**Certainty of the evidence (GRADE)**	**Comments**
**Price**				
Weekly cost per patient	It is uncertain if a cost-feedback approach leads to a difference in costs, because the evidence is inconclusive.	1	Moderate	A cost-feedback approach was associated with an immediate significant reduction in costs for antibiotics. No difference was observed for inhaled corticosteroids.
				Antibiotics showed an increasing trend in costs after the intervention, whereas the approach was associated with a slightly decreasing trend for inhaled corticosteroids.
**Volume**				
-	No studies meeting the inclusion criteria were found	0	-	-
**Availability**				
-	No studies meeting the inclusion criteria were found	0	-	-
**Affordability**				
-	No studies meeting the inclusion criteria were found	0	-	-

*GRADE Working Group grades of evidence

**High** = This research provides a very good indication of the likely effect. The likelihood that the effect will be substantially different^†^ is low.

**Moderate** = This research provides a good indication of the likely effect. The likelihood that the effect will be substantially different^†^ is moderate.

**Low** = This research provides some indication of the likely effect. However, the likelihood that it will be substantially different^†^ is high.

**Very low** = This research does not provide a reliable indication of the likely effect. The likelihood that the effect will be substantially different^†^ is very high.

† Substantially different = a large enough difference that it might affect a decision.

There was no evidence on the impact of this intervention subtype for the outcomes volume, availability or affordability, because these outcomes were not included in the study.

**Disclosure and control of prices along the supply chain** Moodley et al. examined the impact of a national measure that introduced a transparent pricing system in the private market, in the context of the South African Single Exit Price (SEP) policy [22,23]. In an attempt to reduce medicine costs, the 2004 SEP ensures that there is a fixed price for all private sector prescription medicines sold by the manufacturers to distributors and dispensers in the country. The SEP must be publicly disclosed and is composed of the weighted average of the sales price, the logistics fee and value-added taxes, and determined by the manufacturers themselves. Simultaneously, all bonuses, discounts and sampling of medicines were removed. This was complemented with a regulated maximum percentage annual increase and regulation of dispensing fees at retail level. The disclosed prices are made available on the South African Medicine Price Registry website.

The study included 50 medicines within three samples (a ‘global core’ for international comparison, a ‘regional core’ for items important in the region, and a ‘supplementary list’ for products of local importance) as per the WHO/HAI (i.e. Health Action International) methodology. It reports on the prices of medicines paid for by the patient, obtained from dispensing files and claims data. Medicine prices in retail pharmacies across all three samples were reduced immediately following the SEP policy, for both originator and generic medicines (Table 4). Mean reduction was greater for generics. Global core percentage price reduction ranged from 2.45% to 9.12% for originator medicines and 18.50% to 91.52% for generics; regional core reduction was 1.77% to 42.17% for originators and -0.70% to 78.03% for generics; supplementary list price reduction was 11.68% to 55.86% for originators and 9.78% to 78.49% for generics. A (significant) negative change in trend implying continued benefit on patient prices was observed in 26 out of 50 originator medicines and 23 out of 73 generic medicines. The impact of this intervention subtype on the outcomes volume, availability or affordability was not studied.

**Table 4:** Summary of findings of policies promoting public price disclosure.

**Public price disclosure compared to no public price disclosure**				
**Medicines:** 50 medicines originator medicines and corresponding generic medicines divided over a Global and Regional Core, and supplementary lists based on WHO/HAI survey methodology				
**Settings:** South African private sector				
**Intervention:** Price disclosure at the national level (Single Exit Price)				
**Comparison:** No policy				
**Outcomes**	**Impacts**	**No. of studies**	**Certainty of the evidence (GRADE)**	**Comments**
**Price**				
Medicine price	The Single Exit Price policy may be effective in reducing prices of originator and generic medicines immediately after implementation. Benefits may be sustained in originator medicines, whereas long term effects of the Single Exit Price policy on generic medicines may be variable.	1	Low	Medicine prices in all samples (global core, regional core, supplementary list) were reduced immediately following the SEP policy for both generic and originator medicines. Mean reduction was greater for generics.
				Continued benefit on medicine prices through a negative change in trend was observed in approximately half of the originator medicines and a third of the generic medicines.
**Volume**				
-	No studies meeting the inclusion criteria were found	0	-	-
**Availability**				
-	No studies meeting the inclusion criteria were found	0	-	-
**Affordability**				
-	No studies meeting the inclusion criteria were found	0	-	-

*GRADE Working Group grades of evidence

**High** = This research provides a very good indication of the likely effect. The likelihood that the effect will be substantially different^†^ is low.

**Moderate** = This research provides a good indication of the likely effect. The likelihood that the effect will be substantially different^†^ is moderate.

**Low** = This research provides some indication of the likely effect. However, the likelihood that it will be substantially different^†^ is high.

**Very low** = This research does not provide a reliable indication of the likely effect. The likelihood that the effect will be substantially different^†^ is very high.

† Substantially different = a large enough difference that it might affect a decision

## 4 Discussion

Following extensive searches, we found only two studies assessing an intervention promoting price transparency in a manner sufficiently robust for inclusion in this review. It is worth noting that the SEP, while introducing transparency in the private market, also included aspects of price control other than price transparency. With that, evidence on measures that exclusively promote price transparency is even more limited. Nevertheless, the results show that the majority of patient prices of both originator and generic medicines were reduced following a national measure that introduced transparency on the level of the manufacturer. Not only did this policy achieve the intended price reduction, it has also improved accountability of manufacturers through mandatory price disclosure. Findings on the impact of cost-feedback approaches to prescribers are considered inconclusive, due to inconsistent results for different therapeutics. Information about the effects on volume, availability or affordability is currently missing for all transparency initiatives. The 2020 WHO Guideline on Country Pharmaceutical Pricing Policies suggests that countries improve the transparency of pricing and prices, informed by the limited research evidence and additional qualitative information that was considered [14]. These considerations include the notion that transparent pricing or prices could serve multiple purposes, including increased citizen engagement and facilitating other pricing policies such as external reference pricing.

The 2015 WHO Guideline on Country Pharmaceutical Pricing Policies did not yet include policies promoting price transparency in its scope [13]. Despite considerable attention for price transparency measures on the international stage since then, this was not reflected in the amount of robust evidence currently available. Similarly, a recent scoping review [9] on countries’ price transparency initiatives, with a somewhat broader setup that included other study designs as well, confirms that there is limited robust evidence on price transparency policies. This scoping review identified 12 studies, none of which would have been considered eligible for our systematic review. A WHO Technical Report on the pricing of cancer medicines [8] again confirmed that the amount of strong evidence is limited. The small number of studies reporting on the effectiveness of price transparency measures may be due to the complexity inherent to performing this research [31]. At the same time, price transparency measures are currently not common practice, which further contributes to the lack of studies of their real-world effectiveness.

The WHA's resolution to advance the transparency of markets for pharmaceuticals was considered controversial [3,32]. Although the large majority of WHO Member States considered price transparency measures to be key in achieving better access to price data and universal health coverage (UHC), the resolution was strongly contested by several countries. These countries argued that the assessment of potential negative implications of price transparency measures had been insufficient. Specifically, concerns were expressed about the impact of the resolution on developing countries, as improved transparency may threaten differential pricing arrangements [32]. The controversy that the resolution triggered reflects the paradoxical situation of price transparency measures. Without compelling evidence on the impacts of price transparency measures, countries may be cautious to conform to the resolution and implement transparency initiatives. However, without the implementation of novel transparency measures to inform new research, the opportunities for high quality research on the effectiveness of transparency interventions are limited. This ‘Catch-22’ appears to be borne out in the volume of literature identified in this systematic review. Despite this paradox, the resolution may inspire novel policies promoting price transparency to be implemented, which may present new opportunities for research.

The strengths of our systematic review include the use of a rigorous methodological approach, following a pre-defined protocol [17]. We used a sensitive search strategy containing a wide range of terms, designed to retrieve both records that referred to non-specific pharmaceutical pricing policies as well as to price transparency measures specifically. Furthermore, we performed an extensive database search and searched the grey literature, as well as used supplementary search approaches such as checking relevant reference lists and contacting experts. This search strategy reduced the risk of missing potentially relevant studies. Risk of bias and strength of the evidence base were assessed using a validated guideline [19,20] and were determined in duplicate to minimize bias and error.

Our study has several limitations. As noted before, the search resources included grey literature sources. Although important to include such resources, many of the databases have very limited search and exporting functionalities. For those resources, we had to use a more limited range of search terms. This pragmatic search approach is a limitation of the search methods, but should be seen within the wide range of search approaches described above. Another limitation might arise from the heterogeneity of price transparency measures, which may often be interwoven with other pricing policies or which may not be described as a transparency measure by the authors of the study. To minimize this limitation, a standard systematic review approach of using a highly sensitive search approach was used with a broad definition of price transparency policies and search terms, which would identify all types of transparency measures were used. Nevertheless, there is always the chance of missing relevant studies. However, we note that experts in the field were consulted to mitigate this risk. Additionally, the scoping review on transparency measures mentioned earlier, did not identify any studies that we had missed [9]. Finally, due to the nature of policy research, no randomized controlled trials were available to inform on the effectiveness of price transparency measures. Therefore, the certainty of the evidence is lessened due to the use of strong yet quasi-experimental study designs.

The evidence identified on price transparency measures may be limited in applicability, despite its broad relevance in both high- and low-income economies [33,34,35]. The study by Langley et al. [30] focused on two groups of therapeutics only, one of which being antibiotics. The prescription of antibiotics in a high-income setting is expected to be highly regulated and guided by antibiotic susceptibility, so results may not be applicable to other therapeutics. As price transparency initiatives are believed to be promising in a broad range of medicine groups including innovative, anticancer, and other high-priced medicines [35,36], future research should examine the effects of transparency measures in other medicine classes before extrapolating these results. Furthermore, this study was set in a high-income economy that generally requires no co-payment by patients. While these results may be applicable to similar settings, generalizability to healthcare systems in which patients’ ability to pay could influence physician's prescribing behavior is challenging. Similarly, the SEP introduced uniformity in the private market through a transparent pricing system with fixed prices, with the final goal of reducing pharmaceutical expenditures. These results may inform design of policies with similar objectives, but do not immediately apply to other price transparency challenges such as the disclosure of R&D costs. Finally, the overall evidence was limited to only two studies and may not reflect the broad scope that price transparency measures could include. The generalizability of our results to other healthcare systems should therefore be viewed with consideration to context, until such a time when more evidence has been produced. Despite these limitations, this first systematic review is a first step in informing national and regional governments and other policy-makers such as hospital boards or insurance providers on effective policies for managing the prices of pharmaceuticals using transparency measures.

Although opportunities for research on transparency measures seem to be limited due to a ‘Catch-22’ dilemma, it is crucial that when such opportunities do present, efforts of policy-makers and researchers are coordinated. This will help to ensure the collection of data for adequate monitoring of these policies. The conduct of small pilots may help to increase opportunities for evidence generation on the one hand and overcome the reluctance of policy-makers on the other. These future studies should focus on all levels that transparency measures may occur in, and not only on medicine prices or expenditures, but likewise on outcomes such as the volume, availability and affordability of medicines. There should also be a particular focus on unintended and potentially harmful effects of these policies, both in high- as well as in low-income settings. Additionally, the limited amount of evidence currently available is insufficient to elucidate what contextual factors and implementation strategies may influence the effects of such policies, and should be the object of further study.

## 5 Conclusion

In conclusion, the lack of quantitative and comparative evidence assessing the impact of policies promoting price transparency is a clear call for further research. Collaborative pilots involving both national governments and researchers could help to align their interests and overcome the current inertia in evidence development. Additional evidence is needed to confirm the impact of a wide range of transparency measures on the management of medicine prices in countries all over the world. The evidence that is currently available, although from a single study, indicates that a national measure introducing price transparency along the supply chain may be effective in managing medicine prices.

## Role of the funding source

This systematic review was commissioned and funded by the World Health Organization [HQ/EMP/2019/002, 2019] under a grant from the United Kingdom Department for International Development (DFID). The review was part of the process for developing the 2020 WHO Guideline on Country Pharmaceutical Pricing Policies. The WHO secretariat and its advisors provided technical support for formulating the review protocol, performing specific searches on government websites, and translating several non-English publications. WHO, its advisors and DFID had no role in data analysis or interpretation. The views and opinions expressed in this article are those of the authors and do not necessarily reflect the official policy or position of the WHO or DFID.

## Declaration of Competing Interest

This systematic review was commissioned and funded by the World Health Organization.

## Appendix Supplementary materials

Image, application 1
