# Supplementary material for: Evidence on the effectiveness of policies promoting price transparency - A systematic review
Source: Health Policy. 2023 Aug;134:104681. doi: 10.1016/j.healthpol.2022.11.002 (PMC10357344; doi:10.1016/j.healthpol.2022.11.002)
Supplement: Supplementary file 1 [file mmc1.docx]

Appendix 1

***Table S1* description and interpretation of risk of bias domains as applied in the systematic review (1)**

| **Bias domain** | **Explanation** |
| --- | --- |
| **Bias domains specific to interrupted time series and repeated measures studies only** | |
| **Intervention independent** | Low risk” if there are compelling arguments that the intervention occurred independently of other changes over time and the outcome was not influenced by other confounding variables/historic events during study period. If Events/variables identified, note what they are. “High risk” if reported that intervention was not independent of other changes in time. |
| **Appropriate analysis** | “Low risk” if data were analyzed appropriately e.g. if autoregressive integrated moving average (ARIMA) models were used OR time series regression models were used to analyze the data and serial correlation was adjusted/tested for OR reanalysis performed. “High risk” if the outcomes were not analyzed appropriately. “Unclear risk” if not specified in the paper. |
| **Pre-specified shape of effect** | “Low risk” if point of analysis is the point of intervention OR a rational explanation for the shape of intervention effect was given by the author(s). Where appropriate, this should include an explanation if the point of analysis is NOT the point of intervention. “High risk” if it is clear that the condition above is not met. |
| **Intervention to affect data collection** | “Low risk” if reported that intervention itself was unlikely to affect data collection (for example, sources and methods of data collection were the same before and after the intervention). “High risk” if the intervention itself was likely to affect data collection (for example, any change in source or method of data collection reported). |
| **Bias domains applicable to all study types** | |
| **Incomplete outcome data** | “Low risk” if missing outcome measures were unlikely to bias the results (e.g. the proportion of missing data was similar in the intervention and control groups/pre- and post-intervention periods or the proportion of missing data was less than the effect size i.e. unlikely to overturn the study result). “High risk” if missing outcome data was likely to bias the results. “Unclear risk” if not specified in the paper (not assuming 100% complete data unless stated explicitly). |
| **Knowledge of allocated intervention** | “Low risk” if the authors state explicitly that the primary outcome variables were assessed blindly, or the outcomes are objective. Primary outcomes are those variables that correspond to the primary hypothesis or question as defined by the authors. “High risk” if the outcomes were not assessed blindly. Score “Unclear risk” if not specified in the paper. |
| **Selective outcome reporting** | “Low risk” if there is no evidence that outcomes were selectively reported (e.g. all relevant outcomes in the methods section are reported in the results section). “High risk” if some important outcomes are subsequently omitted from the results. “Unclear risk” if not specified in the paper. |
| **Other bias** | “Low risk” if there is no evidence of other risk of biases. |

**References**

1. Cochrane Effective Practice and Organisation of Care. Suggested risk of bias criteria for EPOC reviews. Cochrane Effective Practice and Organisation of Care; 2017. Available from: https://epoc.cochrane.org/sites/epoc.cochrane.org/files/public/uploads/Resources-for-authors2017/suggested_risk_of_bias_criteria_for_epoc_reviews.pdf [accessed 01/04/2021]

Appendix 2

The Cochrane Effective Practice and Organisation of Care (EPOC) guidance for the development of GRADE and Summary of Findings tables outlines the process of GRADE assessment of observational evidence. The GRADE assessment is based on five domains: risk of bias, inconsistency, indirectness, imprecision, and “other”. In this appendix, we clarify the interpretation of these assessment domains.

***Table S2* Description and interpretation of GRADE assessment criteria**

| Domain | Definition by EPOC (1) | Interpretation and adaptation |
| --- | --- | --- |
| Risk of bias | As outlined in appendix 1 | |
| Inconsistency (2) | Inconsistency refers to an unexplained heterogeneity of results. GRADE suggests rating down the quality of evidence if large inconsistency in study results remains after exploration of a priori hypotheses that might explain heterogeneity.  Judgment of the extent of heterogeneity is based on similarity of point estimates, extent of overlap of confidence intervals, and statistical criteria including tests of heterogeneity and I^2^. | As the study types, outcomes and analyses methods tend to vary, we considered inconsistency to indicate the directionality of evidence.  In cases where a point estimate of effect was not statistically significant, the directionality is considered regardless of the precision of the estimate. |
| Indirectness (3) | Includes consideration of   - Indirect (between study) comparisons - Indirect (surrogate) outcomes - Applicability (study populations, interventions or comparisons that are different than those of interest) | The original meaning was consistent with the purposes of the present review. |
| Imprecision (4) | Includes consideration of whether the recommendation would differ, if the true effect would lie at either extreme of the confidence interval. | Analogously, we considered the precision around the estimate of the effect. |
| Other | N/A | Other sources of bias may be related to any features of the study not captured by the points above, or by the risk of bias assessment.  In particular, we considered external validity (generalisability of the evidence) to be of relevance. |

**References**

1. Cochrane Effective Practice and Organisation of Care. EPOC Worksheets for preparing a Summary of Findings (SoF) table using GRADE. Cochrane Effective Practice and Organisation of Care; 2017. Available from: https://epoc.cochrane.org/sites/epoc.cochrane.org/files/public/uploads/Resources-for-authors2017/worksheets_for_preparing_a_summary_of_findings_using_grade.docx [accessed 01/04/2021]
2. Guyatt GH, Oxman AD, Kunz R, Woodcock J, Brozek J, Helfand M, et al. GRADE guidelines: 7. Rating the quality of evidence--inconsistency. J Clin Epidemiol. 2011; 64(12): 1294-302. DOI: 10.1016/j.jclinepi.2011.03.017.
3. Guyatt GH, Oxman AD, Kunz R, Woodcock J, Brozek J, Helfand M, et al. GRADE guidelines: 8. Rating the quality of evidence--indirectness. J Clin Epidemiol. 2011; 64(12): 1303-10. DOI: 10.1016/j.jclinepi.2011.04.014.
4. Guyatt GH, Oxman AD, Kunz R, Brozek J, Alonso-Coello P, Rind D, et al. GRADE guidelines 6. Rating the quality of evidence--imprecision. J Clin Epidemiol. 2011; 64(12): 1283-93. DOI: 10.1016/j.jclinepi.2011.01.012.

Appendix 3

***Table S3* Certainty assessment (GRADE) of evidence for cost-feedback approaches to prescribers**

| **No of studies** | **Design (number)** | **Risk of bias** | **Inconsistency** | **Indirectness** | **Imprecision** | **Other** | **Certainty**  **(overall score)** |
| --- | --- | --- | --- | --- | --- | --- | --- |
| **Outcome:** **Price** | | | | | | | |
| 1 (1) | ITS (I) | Low risk (0) | No serious inconsistency (0) | No serious indirectness (0) | No serious imprecision (0) | Study design (+1) | **Moderate**  ⊕⊕⊕○ |
| **Outcome: Volume** | | | | | | | |
| **Outcome:** **Availability** | | | | | | | |
| **Outcome:** **Affordability** | | | | | | | |

**References**

1. Langley T, Lacey J, Johnson A, Newman C, Subramanian D, Khare M, et al. An evaluation of a price transparency intervention for two commonly prescribed medications on total institutional expenditure: a prospective study. Futur Healthc J. 2018; 5(3): 198–202. DOI: 10.7861/futurehosp.5-3-198

Appendix 4

***Table S4* Certainty assessment (GRADE) of evidence for policies promoting public disclosure of prices**

| **No of studies** | **Design (number)** | **Risk of bias** | **Inconsistency** | **Indirectness** | **Imprecision** | **Other** | **Certainty**  **(overall score)** |
| --- | --- | --- | --- | --- | --- | --- | --- |
| **Outcome:** **Price** | | | | | | | |
| 1 (2,3) | ITS (I) | Low risk (0) | No serious inconsistency (0) | Serious indirectness  (-1)† | No serious imprecision (0) | Study design (+1) | **Low**  ⊕⊕○○ |
| **Outcome: Volume** | | | | | | | |
| **Outcome:** **Availability** | | | | | | | |
| **Outcome:** **Affordability** | | | | | | | |

†The SEP was associated with serious indirectness, because there appear to be multiple aspects of price control other than transparency.

**References**

1. Moodley R, Suleman F. The impact of the single exit price policy on a basket of generic medicines in South Africa, using a time series analysis from 1999 to 2014. PLoS ONE. 2019; 14(7):e0219690. DOI: 10.1371/journal.pone.0219690
2. Moodley R, Suleman F. Evaluating the impact of the single exit price policy on a basket of originator medicines in South Africa from 1999 to 2014 using a time series analysis. BMC Health Serv Res. 2019; 19(1) :576. DOI: 10.1186/s12913-019-4403-8
